# EEG dataset from playing Multiplayer Online Battle Arena games in natural settings

**DOI:** 10.1038/s41597-025-05435-5

**Published:** 2025-07-02

**Authors:** Hong-Zhi Li, Jia-Jia Yang, Zhen Lv, Li-Yang Wan, Wo Wang, Da-Qi Li, Dong-Dong Zhou, Li Kuang

**Affiliations:** 1 Mental Health Center, University-Town Hospital of Chongqing Medical University, Chongqing, China; 2 Mental Health Education and Counseling Center, Chongqing jianzhu College, Chongqing, China; 3 Psychiatric Center, The First Affiliated Hospital of Chongqing Medical University, Chongqing, China

**Keywords:** Cognitive neuroscience, Psychology and behaviour

## Abstract

Mobile Multiplayer Online Battle Arena (MOBA) games have emerged as one of the most popular gaming genres, yet the underlying neurophysiological mechanisms contributing to their addictive potential remain unclear. In this study, 23 participants played six real matches of Honor of Kings while synchronized 64-channel EEG recordings were conducted. We provide EEG data collected during gameplay, alongside corresponding video recordings. Additionally, we developed an experimental protocol that accurately marks the timestamps of player kills and deaths within the EEG data. This allows for an investigation of neurophysiological responses to kills and deaths at a millisecond-level time scale within actual MOBA gameplay. Furthermore, we include resting-state EEG data recorded in both eyes-open and eyes-closed conditions, as well as participants’ demographic information and scores related to gaming addiction, impulsivity, and emotional regulation. This dataset aims to contribute to the understanding of neurophysiological responses in natural MOBA gaming environments, providing open access resources with high ecological validity.

## Background & Summary

Online gaming has become a significant form of entertainment for a vast number of internet users, encompassing a wide variety of genres. Among these, Multiplayer Online Battle Arena (MOBA) games have gained immense popularity due to their competitive fairness and real-time engagement^1^. With the continuous advancement of mobile technology, users can now access MOBA games conveniently on portable devices, leading to a growing user base, particularly in Asia^2^. However, excessive engagement with gaming can result in Internet Gaming Disorder (IGD), significantly impacting individuals’ academic performance, daily life, and interpersonal relationships. Recent studies indicate that the prevalence of IGD stands at 13.4%, with approximately 49.4% of cases associated with MOBA games^3^. This underscores the importance of further investigating the mechanisms underlying gaming and gaming addiction.

Research on the neuroimaging of gaming has been increasing, revealing that gameplay can alter brain structure and function^4–7^. Some studies suggest a negative impact of gaming on cognitive control^8^, while others indicate potential improvements in cognitive function^9,10^. Differential neural responses are observed across various game genres^11^, yet many previous studies have not specifically distinguished between addictive game types, possibly contributing to the heterogeneity observed in IGD research^12,13^. For instance, Na *et al*.^14^ highlighted that different risk factors for addiction exist across gaming genres, suggesting that intervention strategies should consider specific game types^14^. Balhara *et al*.^15^ emphasized the need for clear definitions regarding game types when addressing gaming addiction^15^. Therefore, it is essential for future research to investigate the neurophysiological mechanisms of addiction and intervention strategies specific to particular game genres.

Most existing research has been conducted under experimental conditions, which may limit ecological validity; thus, there remains a lack of studies conducted in natural gaming environments. Some researchers, however, have made meaningful attempts in this area. For example, Long *et al*. (2024) recorded functional near-infrared spectroscopy (fNIRS) while participants played League of Legends, revealing frontal region activations related to key in-game events, influenced by physiological arousal and individual player characteristics^16^. Klasen *et al*. (2020) used functional magnetic resonance imaging (fMRI) during gameplay of Carmageddon, finding that non-violent successes activated the ventral striatum, whereas violent successes specifically activated the dorsal striatum, with subjective game experiences correlating with activation in the putamen and medial prefrontal cortex during violent successes^17^. These neuroimaging studies conducted in real gaming contexts demonstrate that key in-game events can elicit changes in brain activity associated with reward systems and cognitive control. Additionally, Xi *et al*. (2022) proposed a reinforcement-based model of gaming addiction, positing that frequent direct rewards obtained during gameplay positively reinforce gaming behavior, leading to its repetition^18^. Such evidence suggests that IGD research should also focus on key events occurring during real gameplay.

Given the dynamic nature of real gaming, EEG offers a valuable method for observing brain activity in naturalistic settings due to its high temporal resolution. Our dataset features experienced mobile MOBA gamers, from whom resting-state EEG data were collected prior to gameplay. We simultaneously recorded their real-time EEG signals during gameplay and designed an experimental protocol to accurately mark the timestamps of kills and deaths during MOBA game. This approach allows for a higher temporal resolution analysis of players’ neurophysiological responses following key events in real gameplay. Additionally, we assessed participants’ emotional regulation abilities, levels of gaming addiction, and impulsivity through scales, as these psychological traits are closely linked to gaming behavior^19–21^. We believe this dataset will facilitate to explore the relationship between psychological characteristics and neurophysiological responses following key events in gameplay, and a deeper understanding of the neurophysiological responses of MOBA gamers in natural settings.

## Methods

### Overall design

Data collection for this study commenced in April 2023 and concluded in July 2023. Prior to the study, all participants underwent screening for psychiatric disorders using the Mini-International Neuropsychiatric Interview (M.I.N.I)^22^. Participants completed three tasks: (1) scale assessments; (2) resting-state EEG recordings; and (3) EEG recordings during MOBA gameplay. The conduction of the experiment was in accordance with the Declaration of Helsinki. This study was approved by the Ethics Committee of University-Town Hospital of Chongqing Medical University (Approval No. LL-202307).

### Participants

A total of 23 voluntary participants were recruited via online advertisement, comprising 15 males and 8 females, with a mean age of 19.70 years. All participants were right-handed and had a minimum of one year of MOBA gaming experience. Prior to participation, all subjects provided written informed consent after receiving comprehensive explanations of the research procedures. This consent specifically included explicit permission for public sharing of both their EEG data and MOBA gameplay videos for scientific purposes. Importantly, they also assured that their virtual game identifier could not be linked to real-world identities, thus fully protecting participant privacy. Each participant received 100 CNY upon completion of all experimental tasks. For minors under 18 years of age, both the participants and their legal guardians were fully briefed and provided written consent for participation and data sharing.

Exclusion criteria included: (1) a history of head injury or neurosurgery; (2) current or past diagnoses of neuropsychiatric disorders; and (3) chronic physical illnesses. Participants were instructed to ensure adequate sleep in the week leading up to the study and to abstain from psychoactive substances such as tobacco, alcohol, and caffeine.

### Scale assessments

Participants were informed that there were no right or wrong answers to the scale items and were encouraged to respond truthfully. For each scale, instructions were provided to participants, who selected the most applicable option for each item. Any questions were addressed by trained researchers to ensure participants understood the items clearly. The following scales were used:

- Demographic Questionnaire: Demographic data comprised gender, age, height, weight, MOBA gaming rank (e.g., Diamond, Master), and weekly MOBA gaming duration (hours).
- 20-item Internet Gaming Disorder (IGD-20): This scale comprises six factors: salience, mood modification, tolerance, withdrawal, conflict, and relapse, totaling 20 items with a 5-point rating scale (1 = strongly disagree, 5 = strongly agree). Higher scores indicate greater levels of gaming addiction^23,24^.
- Barratt Impulsiveness Scale (BIS-11): This scale includes three subscales: nonplanning impulsiveness, motor impulsiveness, and attentional impulsiveness, each with 10 items rated on a scale from 1 to 4. The Chinese version adjusts the scoring range to 1–5, with higher scores reflecting greater impulsivity^25,26^.
- Difficulties in Emotion Regulation Scale (DERS): Comprising 36 items, this scale assesses six dimensions: non-acceptance of emotional responses, difficulties engaging in goal-directed behavior, impulse control difficulties, lack of emotional awareness, limited access to emotion regulation strategies, and lack of emotional clarity, rated on a 5-point Likert scale. Higher total scores indicate poorer emotional regulation ability^27,28^.

### EEG online recording

**Fig. 1:**
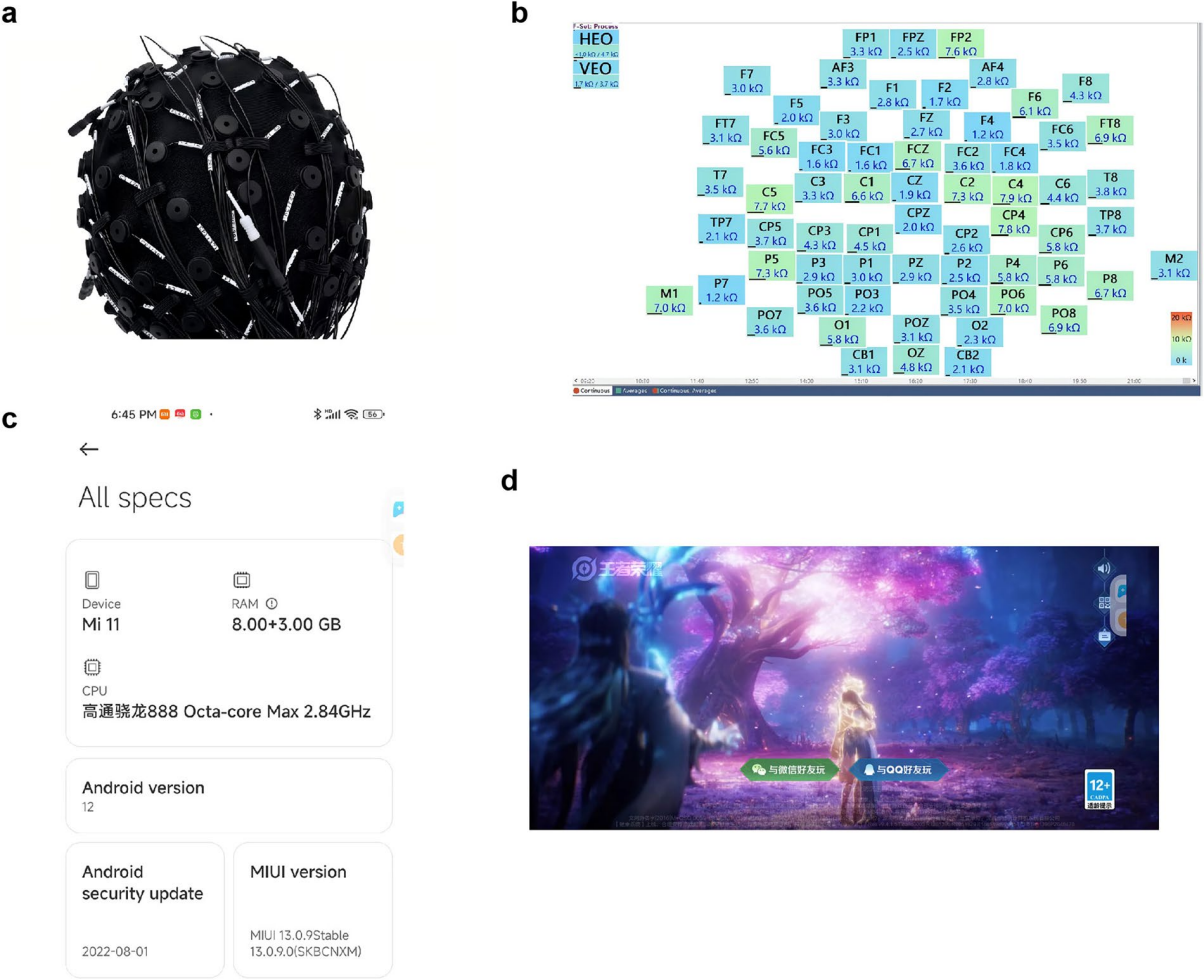
Experimental materials. (**a**) 64-channel electrode cap; (**b**) Electrode distribution and impedance display during an experiment with a participant; (**c**) Specifications of the Xiaomi phone used for the experiment; (**d**) Login interface of the game “Honor of Kings”.

Participants were tested in a quiet room using Curry 8 software and the Neuroscan 64-channel EEG recording system, adhering to the 10–20 international electrode placement system, with electrode resistance maintained below 10 kΩ (Fig. 1a,b). An online reference electrode was positioned at the midpoint between Cz and Cpz, with an online filter range of 0.05–400 Hz and a sampling rate of 1000 Hz.

### Resting-state task

Participants completed a total of 14 minutes of resting-state EEG recording, with 7 minutes collected in the eyes-open condition while fixating on a “+” displayed on a computer screen, and 7 minutes in the eyes-closed condition, remaining awake without falling asleep. During EEG collection, participants were instructed to minimize bodily movement.

### MOBA Game Task

All gameplay involved the MOBA game Honor of Kings (Fig. 1d), developed by Tencent’s TiMi Studio. The game features various modes including 1v1, 3v3, and 5v5. In this study, participants engaged in the 5v5 ranked mode, which involves 10 players divided into two teams. Players control chosen characters to destroy the opposing team’s base while protecting their own, with typical game durations of 10–20 minutes. Victory is achieved by destroying the enemy base or securing a surrender. Players accumulate rank points for victories, with rankings ranging from Bronze to Glory King.

All participants used a uniform Android device (Xiaomi 11 Pro) specifically for this study. The device operated on Android, had 8GB RAM, and a 6.81-inch screen with a resolution of 3200 × 1440 pixels (Fig. 1c). The device utilized a 5 G network for optimal gameplay performance. Each participant logged into their game account, and those typically using Apple devices were provided with a suitable Android account for gameplay. Throughout the experiment, participants played six matches of the MOBA game. Game audio was played aloud to enhance immersion and replicate authentic gameplay conditions. Researchers informed participants to play in their usual manner, emphasizing that their performance would not be evaluated or affect the study. Participants were then instructed to minimize large movements during gameplay and to freely choose their characters based on their preferences and analyses of the game situation.

**Fig. 2:**
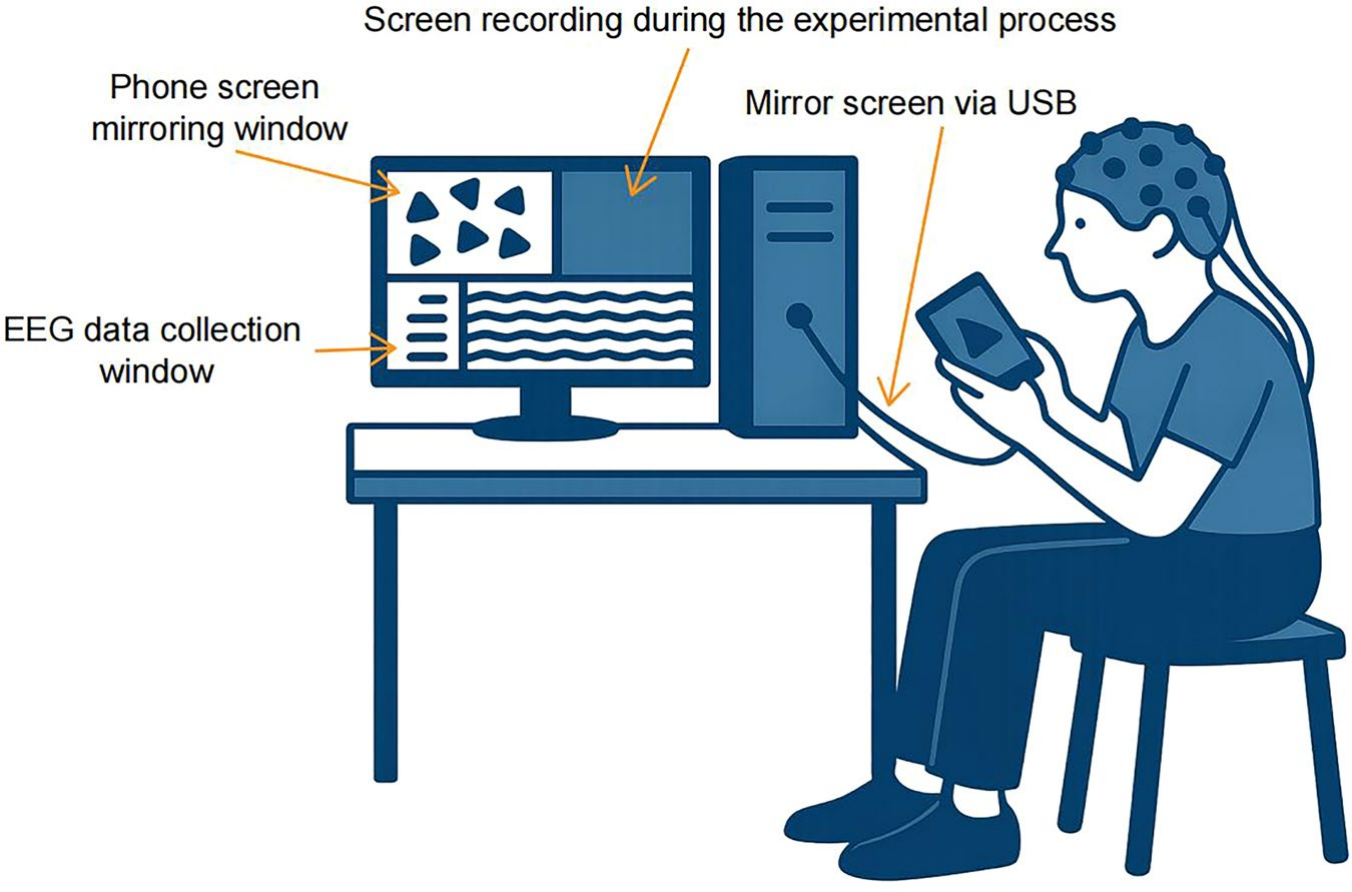
Illustration of simultaneous EEG recording, screen mirroring, and video recording while participants play the MOBA game.

**Fig. 3:**
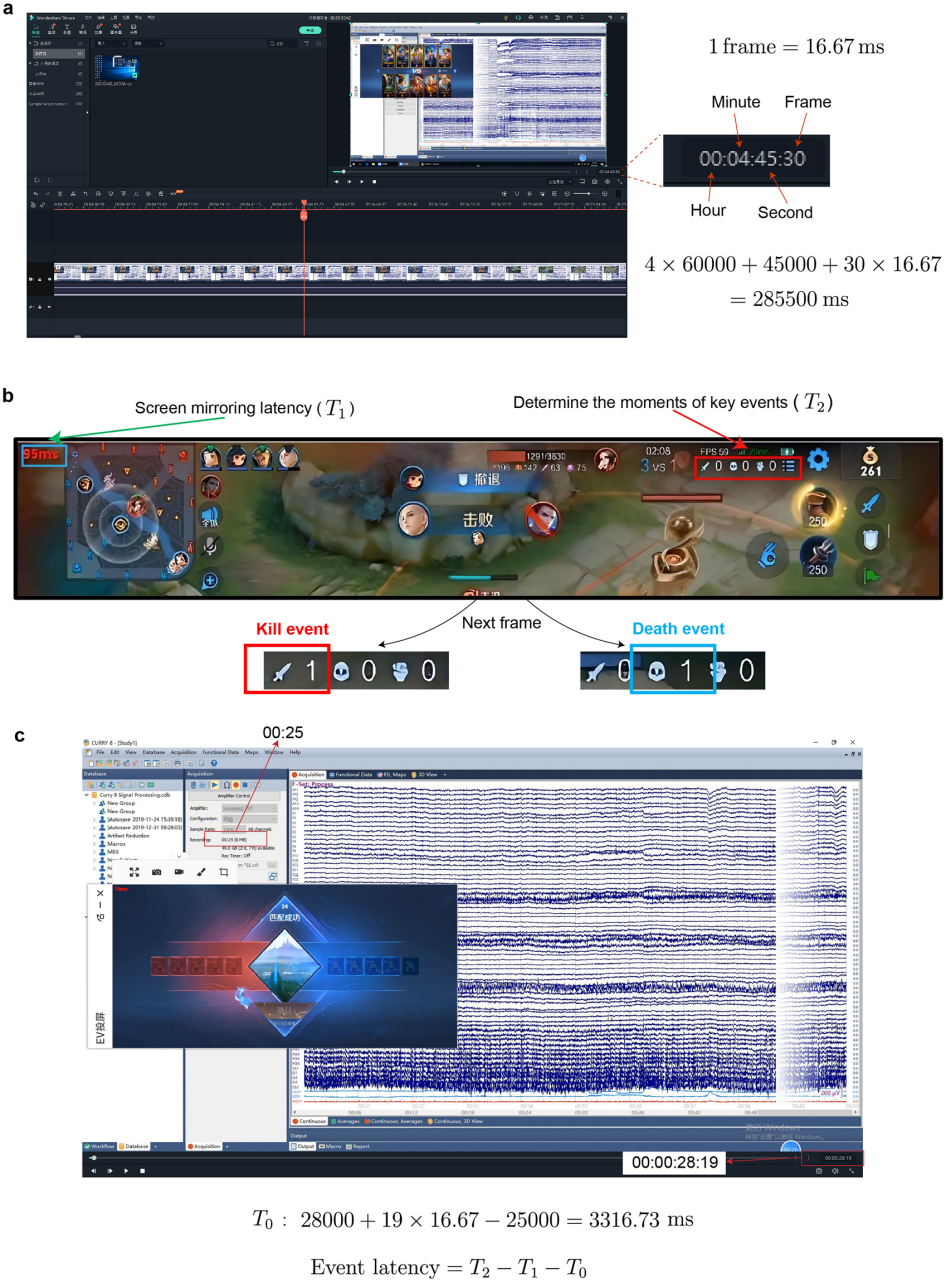
Illustration of determining precise time points for key events. (**a**) Example of the method for calculating precise time points during frame-by-frame video review. (**b**) Method for determining the time points of kills and deaths; (**c**) Establishing the start time for EEG recording.

**Fig. 4:**
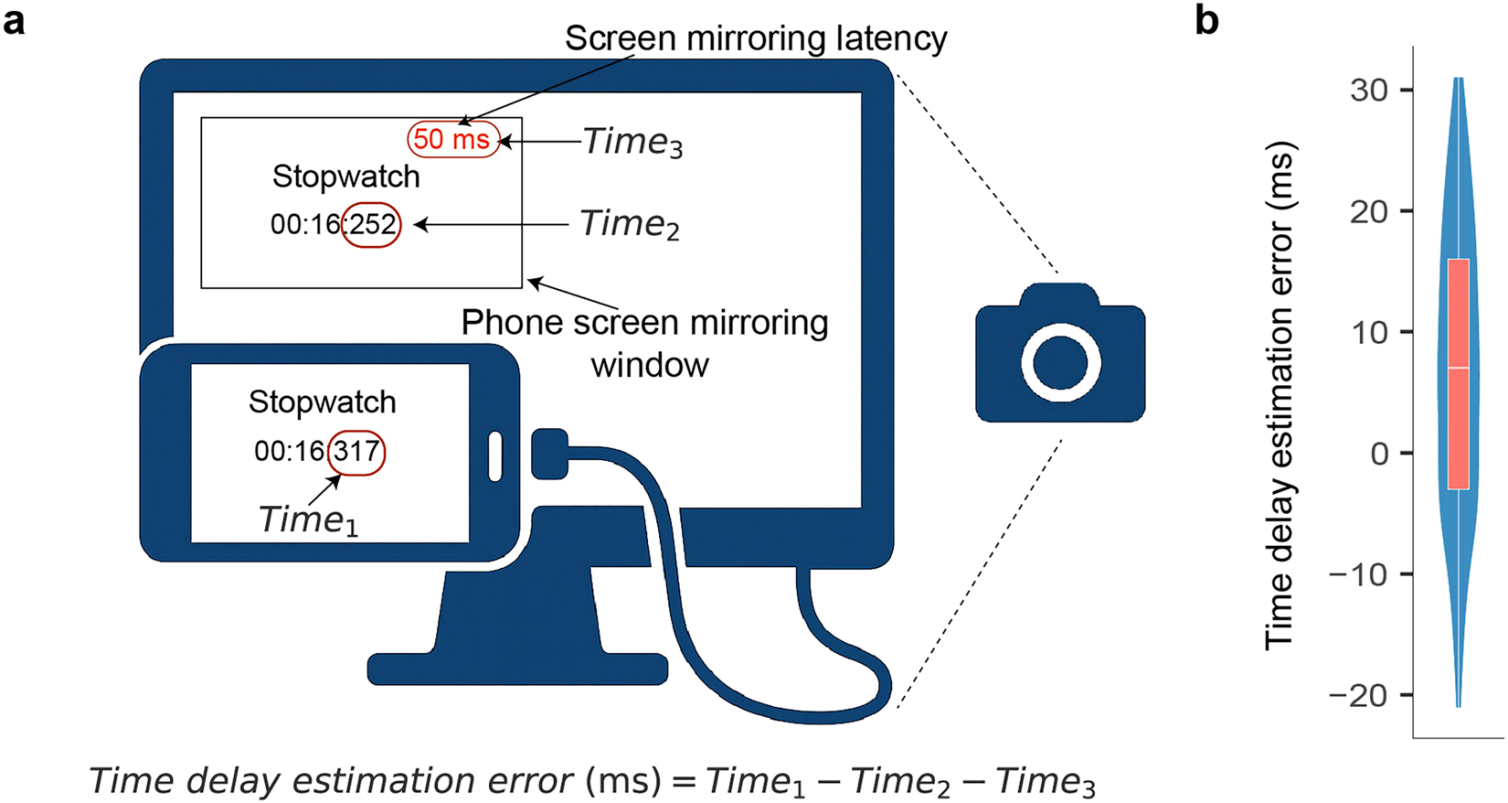
Example of calculating screen mirroring delay error. (**a**) Schematic diagram of the method for estimating screen mirroring delay error; (**b**) Results of 100 calculations of screen mirroring delay error.

The experimental procedures were conducted following the methodologies presented in Figs. 2, 3. The gaming device was connected to the EEG recording computer via USB. The gameplay was displayed on the computer through EVScreenMirror, utilizing USB for screen mirroring with a frame rate of 60 fps. We customized a feature with Hunan Yiwei Information Technology Co., Ltd. that allowed real-time display of the screen mirroring delay in milliseconds in the upper left corner of the computer screen, with an average error of 6.84 ms (Fig. 4). Simultaneously, we used EVCapture to record both the EEG data and the gameplay footage, with a recording frame rate set to 60 fps and saved in AVI format. The collection of EEG data and screen recording concluded after all six matches were completed. After each match, participants were allowed a brief rest before starting the next one, and recordings continued uninterrupted during these breaks.

### Acquisition of critical event timestamps

Post-experiment video analysis was performed by two raters (DDZ and HZL) working independently. Using Wondershare Filmora (Wondershare Technology Co., Ltd.), they examined each video frame (16.67 ms/frame) to objectively identify and record timestamps for all critical in-game events, establishing a reliable event marker dataset. The key events recorded included game start, kills, deaths, game victory, and game defeat. The method for determining the timestamps of these events based on frame-by-frame analysis is illustrated in Fig. 3. We conducted a frame-by-frame comparison to identify the exact moments key events occurred, utilizing the progress bar in the software to convert the specific frames into milliseconds. Due to inherent delays in data transmission when the gameplay was mirrored from the mobile device to the computer, the timestamps for all key events were adjusted by subtracting the mirroring delay to reflect the actual occurrence time of these events.

It is essential to note that the timestamps for the occurrence of key events are based on the video recording. Therefore, we also determined the timestamp marking the beginning of EEG data recording in the video (T_0_). By subtracting T_0_ from the timestamps of key events, we calculated the latencies of these events in the EEG data (Fig. 3). The timestamps for the occurrence of each event were determined as follows: (1) EEG Start Time (T_0_): As illustrated in Fig. 3c, T_0_ was calculated by subtracting the Curry8 recording timestamp from the corresponding video frame time when the temporal counter transitioned in the Curry8. Two independent evaluators derived four T_0_ measurements (pre-first match and post-final match). The modal value was selected as the definitive T_0_ for event latency computations. (2) Game Key Events: The first frame indicating an increase in the numbers for “kills” and “deaths” on the scoreboard determined the timestamps for these events, as illustrated in Fig. 3b. We designated the following markers for key event types in the EEG data: kills were marked as 13; deaths as 14; game start as 66; game victory as 666; and game defeat as 444. The event marker files in TXT format have been made publicly accessible through the OpenNeuro repository under the directory ‘\derivatives\markers’.

## Data Records

All data are available in BIDS format^29,30^, and uploaded to the OpenNeuro site^31^. The main folder of this dataset contains 23 folders—one for each participant—and a derivatives folder that includes pre-processed data and code for reproducing the figures and technical validation^31^. This folder also contains four files: (1) “data-description.json” which describes the dataset; (2) “participants.tsv” containing participant information such as sex, age, MOBA gaming rank (e.g., Diamond, Master), weekly MOBA gaming duration (hours), and scores from IGD-20, BIS-11, and DERS; (3) “participants.json” which details all columns in the “participants.tsv” file; and (4) “README” providing general information about the dataset, including contact details. Each participant’s folder includes EEG data, electrode placements, channels, etc., for both resting and task states during MOBA gameplay.

**Fig. 5:**
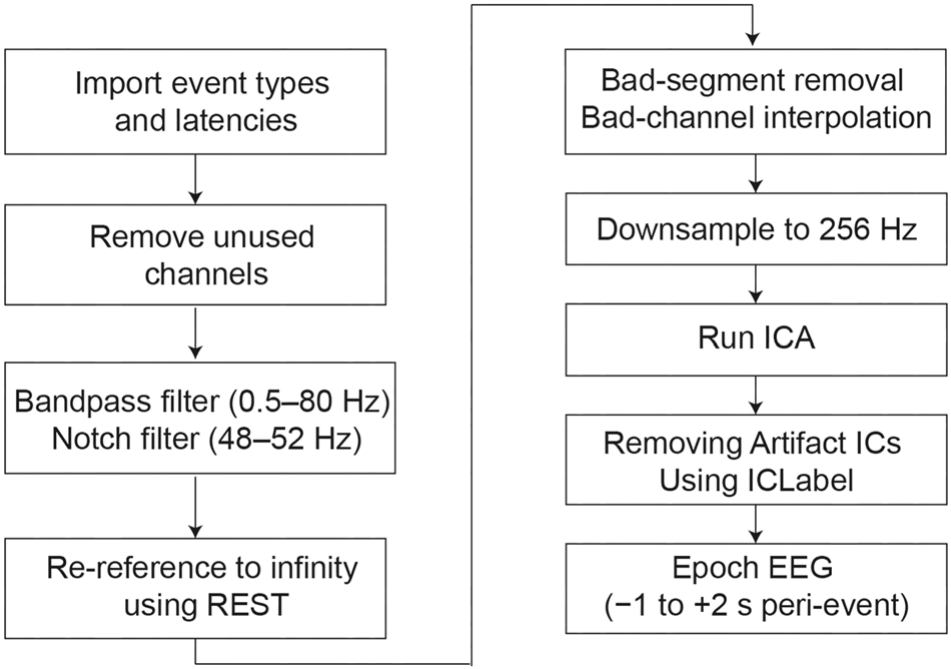
EEG preprocessing pipeline.

The EEG offline processing was performed using EEGLAB 2023 in MATLAB R2019a^32^. First, we imported the key event markers and their associated latencies obtained from video review into the EEG data files, resulting in an EEG file with markers. Second, we removed unused channels, such as ‘M1’, ‘M2’, ‘HEOG’, ‘VEOG’, ‘CB1’, and ‘CB2’. Third, EEG signals were band-filtered between 0.5 Hz and 80 Hz, with a notch filter applied between 48 Hz and 52 Hz to mitigate power frequency interference. Fourth, all EEG signals were re-referenced to an infinity reference using the Reference Electrode Standardization Technique (REST)^33^. Fifth, we manually removed bad segments and interpolated any bad channels, followed by downsampling to 256 Hz and running independent component analysis (ICA). Independent component classification was performed using ICLabel with a probability threshold of 0.7, followed by automated removal of artifact-designated components (ICs)^34^. Subsequently, EEG signals were segmented into epochs time-locked to key gameplay events (kills/deaths), spanning from −1,000 ms pre-event to +2,000 ms post-event. Epochs containing kill-death intervals shorter than 1,000 ms were systematically excluded to avoid temporal overlap contamination. The complete EEG preprocessing pipeline is illustrated in Fig. 5. All preprocessed EEG datasets were stored in the ‘\derivatives\preprocessed’ directory, with the corresponding preprocessing scripts (preprocessing_code.m) archived in the same location for public access.

Additionally, under the path ‘\derivatives\movies’, we provided video recordings of all participants playing the game. Researchers can select time points or intervals of interest during MOBA gameplay based on the methods outlined in this paper for further EEG analysis. The path ‘\derivatives\markers’ contains the latency of key events in each participant’s task-state EEG data, and we have already marked these key events in the ‘sub-**_task-MOBAgame_eeg.eeg’ file.

## Technical Validation

### Reliability analysis

As previously described, two independent researchers (DDZ and HZL) manually annotated key event latencies (i.e., in-game kills and deaths) by reviewing all participants’ gameplay videos. Following the methodology outlined in Fig. 3b, the timestamps of in-game kills and deaths were recorded with absolute consistency between raters. However, potential discrepancies could arise in determining the EEG start time (T_0_).

Four T_0_ values were obtained:

r1b: T_0_ calculated by Rater 1 at the beginning of the experiment.

r1e: T_0_ calculated by Rater 1 at the end of the experiment.

r2b: T_0_ calculated by Rater 2 at the beginning of the experiment.

r2e: T_0_ calculated by Rater 2 at the end of the experiment.

**Fig. 6:**
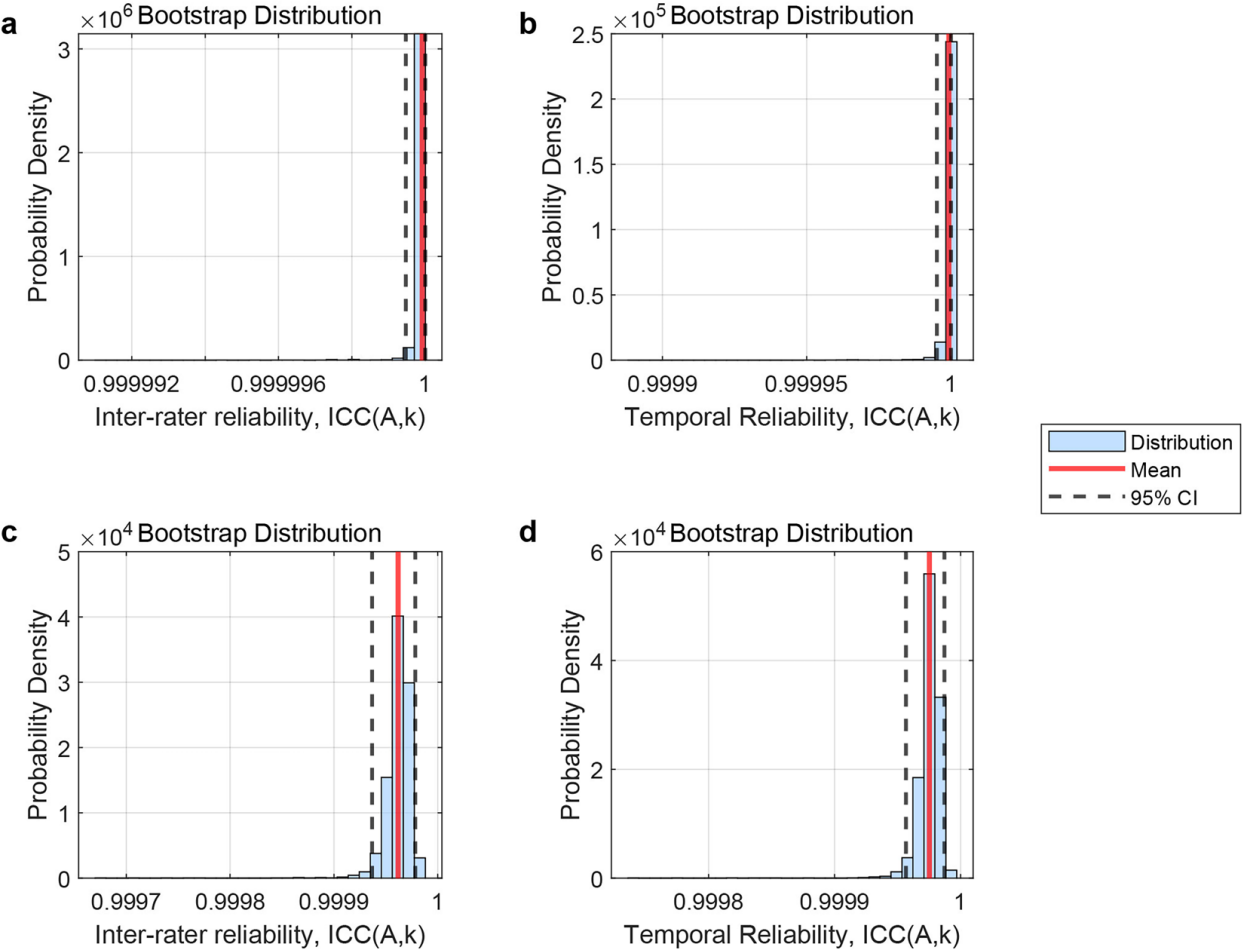
Reliability analysis. (**a**) Inter-rater reliability across all subjects; (**b**) Temporal Reliability across all subjects; (**c**) Inter-rater reliability for each MOBA game round in sub01, sub02, and sub11; (**d**) Temporal Reliability for each MOBA game round in sub01, sub02, and sub11.

Inter-rater reliability was computed by first averaging each rater’s two measurements (r1b/r1e for Rater 1; r2b/r2e for Rater 2) and then calculating the intraclass correlation coefficient (ICC) between raters. Similarly, temporal reliability was assessed by averaging the two raters’ measurements at the beginning (r1b/r2b) and end (r1e/r2e) of the experiment, followed by ICC computation between these two time points. Using bootstrapping (5,000 iterations), both inter-rater and temporal reliability yielded high ICC values of 1.00 (Fig. 6a,b), indicating good consistency in T_0_ determination across raters and experimental phases.

**Fig. 7:**
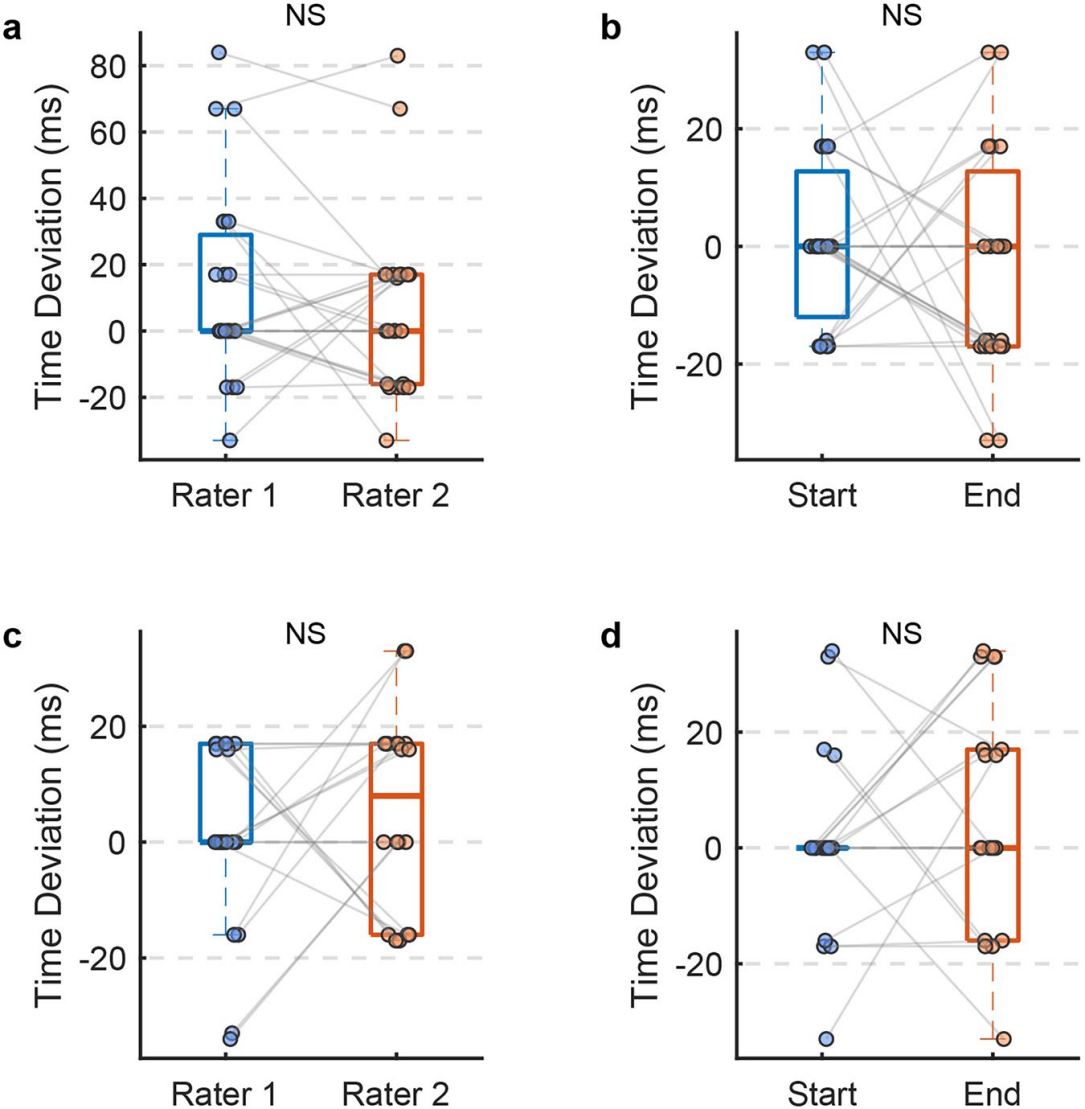
Absolute temporal discrepancies across raters and experimental phases. (**a**) Absolute inter-rater temporal discrepancies across all subjects; (**b**) Absolute temporal discrepancies between the beginning and end of the experiment for all subjects; (**c**) Absolute inter-rater temporal discrepancies for each MOBA game round in sub01, sub02, and sub11; (**d**) Absolute temporal discrepancies between the beginning and end of each MOBA game round in sub01, sub02, and sub11.

We further examined the absolute temporal discrepancies among the four T_0_ measurements (r1b, r1e, r2b, r2e). As illustrated in Fig. 7, three participants (sub01, sub02, sub11) exhibited larger deviations (>50 ms) between T_0_ values obtained at the beginning vs. end of the experiment, whereas all other participants showed highly consistent T_0_ values (absolute differences <35 ms) across both raters and experimental phases. For these three participants, we implemented an adaptive T_0_ recalibration strategy: T_0_ was recalculated before and after each MOBA game, and the final event timestamps were computed separately for each round of game. Both inter-rater reliability (ICC = 1.00) and temporal reliability (ICC = 1.00) were observed for these three participants (Fig. 6c,d). Moreover, the absolute differences of T_0_ values across both raters and experimental phases were less than 35 ms for these three participants (Fig. 7c,d). This validates that our proposed temporal alignment framework achieves both high inter-rater reliability (minimizing observer bias) and robust temporal stability (resistant to signal drift in prolonged EEG recordings). All datasets and analysis routines for reliability assessment have been archived in the ‘\derivatives\Reliability_analysis’ directory to ensure full methodological transparency.

### EEG quality

During EEG preprocessing, an average of 0.48 ± 0.90 channels were interpolated due to excessive noise. Additionally, 20.04 ± 5.03 independent components (ICs) were automatically removed using the ICLabel plugin, primarily reflecting artifacts from ocular (blinks, horizontal eye movements) and muscle activity. Given that participants continuously moved their fingers during gameplay and frequently exhibited orofacial movements (e.g., swallowing, teeth clenching, frowning), myogenic artifacts were notably prevalent.

**Fig. 8:**
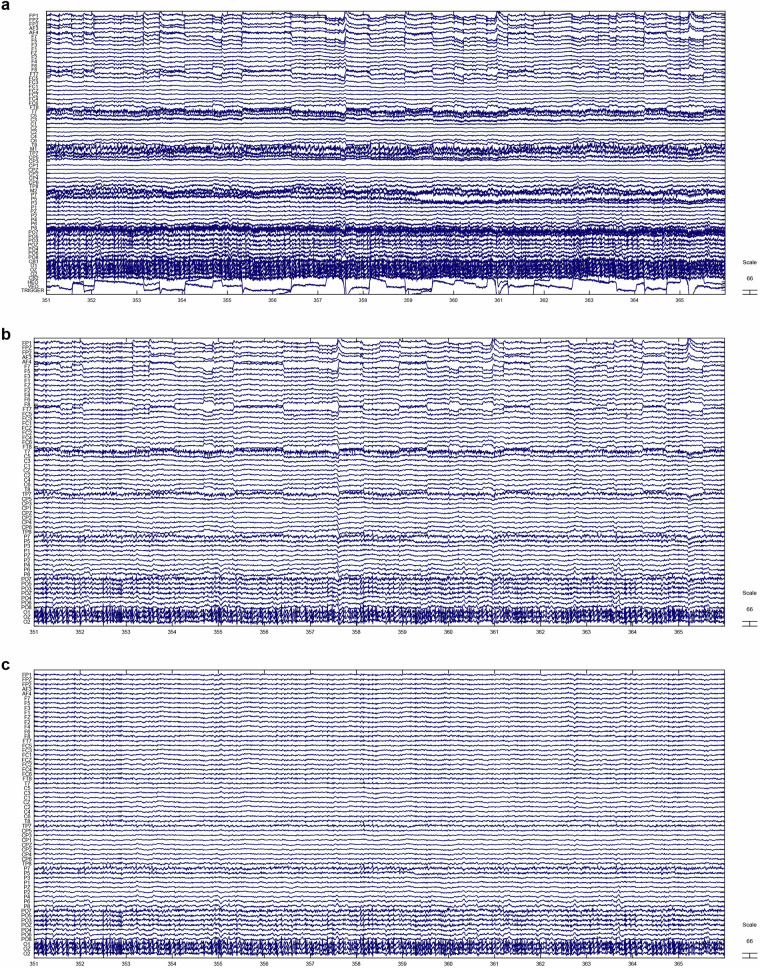
Comparison of raw and preprocessed EEG segments. (**a**) A raw EEG segment from sub01; (**b**) The same EEG segment after preprocessing (0.5–80 Hz bandpass filtering, 48–52 Hz notch filtering, and REST re-referencing); (**c**) The same EEG segment after independent component artifact removal.

Figure 8a presents a representative raw EEG segment from sub01. Following preprocessing steps—including bandpass filtering (0.5–80 Hz), 50 Hz notch filtering, and REST re-referencing—significant noise reduction is observed (Fig. 8b). Further refinement through IC-based artifact removal demonstrates robust suppression of key artifact sources: ocular artifacts (blinks, saccades) and muscle-related artifacts (Fig. 8c). These results confirm that our preprocessing pipeline robustly mitigates major artifact sources while preserving neural signals of interest.

### P300 component validation

Based on the preprocessed EEG data, we applied a 30 Hz low-pass filter and segmented the signals into epochs spanning −200 ms to + 1000 ms relative to each key event onset, followed by baseline correction (−200 to 0 ms). The preprocessed EEG datasets for kills and deaths were stored in ‘\derivatives\preprocessed\13’ and ‘\derivatives\preprocessed\14’, respectively.

**Fig. 9:**
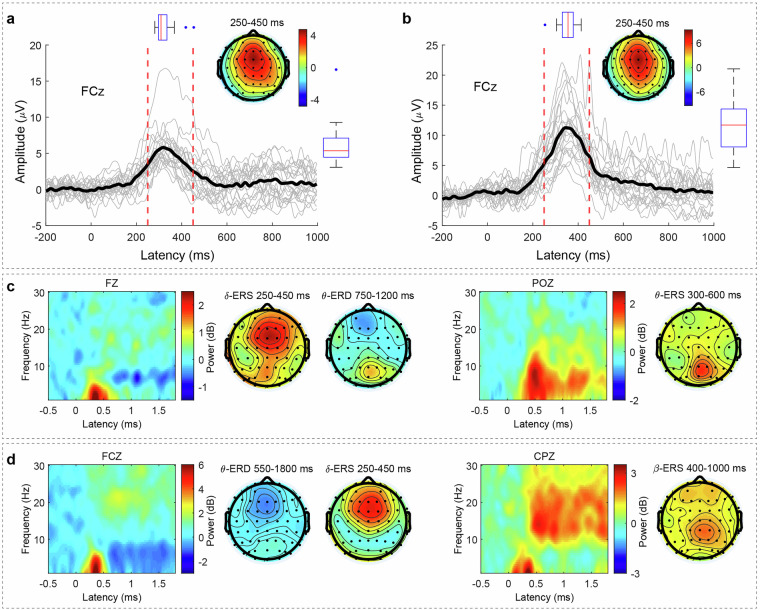
P300 and time-frequency responses following in-game events. (**a**) ERP waveforms following an in-game kill event; (**b**) ERP waveforms following an in-game death event; (**c**) Time-frequency responses following an in-game kill event; (**d**) Time-frequency responses following an in-game death event.

We anticipated robust P300 component emergence within the 250–450 ms temporal window, and the results demonstrated obvious P300 peak activity within this timeframe following both kill and death events during gameplay (Fig. 9a,b). These findings indicate that our experimental methodology and data acquisition protocols successfully captured neural responses associated with in-game events, thereby establishing strong ecological validity. All ERP analysis scripts and processed datasets are organized under the directory path: ‘\derivatives\ERP_analysis’.

### SNR analysis

We calculated the signal-to-noise ratio (SNR) for each channel by defining the pre-stimulus 200 ms EEG interval as the noise baseline and the post-stimulus 200–1000 ms period as the signal window, using the following formula:

$${{\rm{SNR}}}_{{\rm{dB}}}=10{\log }_{10}\left(\frac{{\mathrm{var}}_{{\rm{signal}}}}{{\mathrm{var}}_{{\rm{noise}}}}\right)$$

**Fig. 10:**
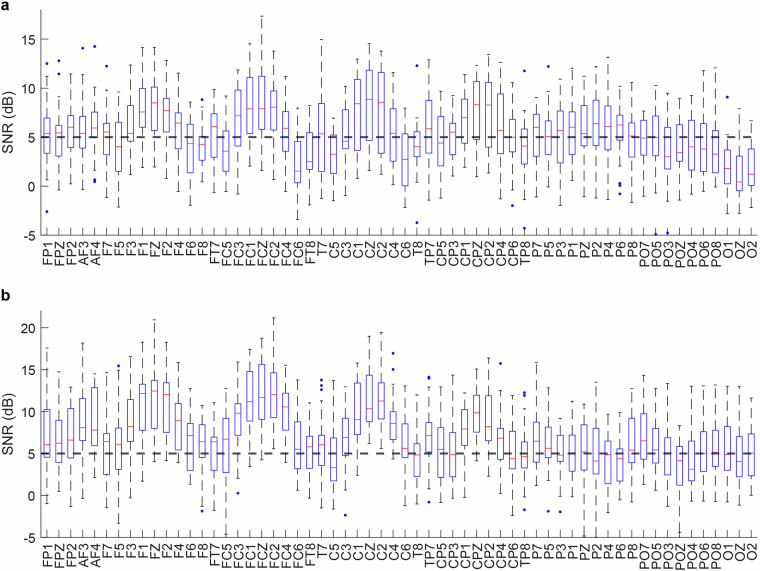
SNR across all channels. (**a**) SNR during in-game kill epochs; (**b**) SNR during in-game death epochs.

The analysis revealed superior SNR values in frontal-central electrodes (Fz, FCz, Cz), while other channels exhibited relatively lower SNR (Fig. 10). This SNR reduction in peripheral channels likely stems from pronounced myogenic artifacts generated during gameplay, including (1) sustained finger movements controlling the interface, (2) ocular artifacts from intense visual fixation and saccades, and (3) orofacial muscle activity (swallowing, jaw clenching, and brow movements). All SNR computation scripts and derivative data have been archived in the ‘\derivatives\SNR’ directory, ensuring full reproducibility of these electrophysiological quality metrics.

### Time-frequency analysis

We conducted time-frequency analysis using short-time Fourier transform (STFT) with a 400-ms Hanning window across the 1–45 Hz frequency range to examine neural oscillatory dynamics following in-game events, applying baseline correction based on the pre-stimulus interval (−800 to −200 ms). Our analysis revealed prominent delta-band event-related synchronization (ERS) and theta-band event-related desynchronization (ERD) following both kill and death events. Similarly, theta-ERS was observed over parieto-occipital regions after in-game kills, while beta-ERS emerged over centro-parietal regions following in-game deaths (Fig. 9c,d).

### Correlation analyses

**Fig. 11:**
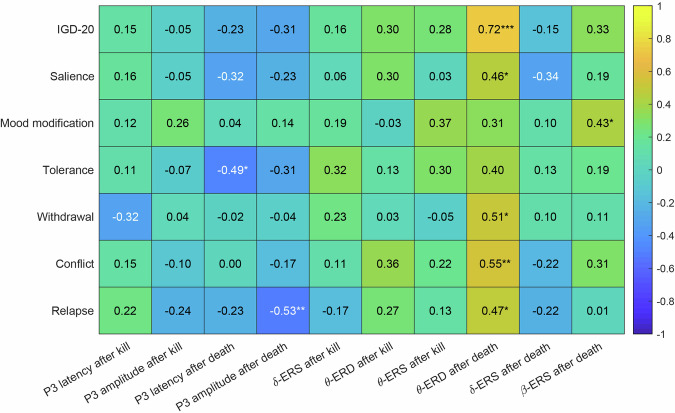
Correlation analyses between P300 features, time-frequency responses, and IGD-20 total and subscale scores.

Initial correlation analyses showed significant positive association between total IGD-20 score and post-death theta-ERD, with full results in Fig. 11 and analysis code at ‘\derivatives\correlation_analysis’.

## Usage Notes

This dataset has multiple potential applications in cognitive neuroscience and IGD-related research, including: (1) Investigating EEG characteristics during real MOBA gameplay (e.g., power spectral analysis, functional connectivity, microstate analysis) compared to resting states; (2) Analyzing neural responses to critical events (kills and deaths) during gameplay at a millisecond time scale (e.g., event-related potentials, time-frequency analysis); (3) Allowing researchers to annotate their own time points or intervals of interest based on recorded gameplay videos to explore corresponding EEG characteristics; (4) Examining correlations between the aforementioned objective EEG features and the psychological characteristics of MOBA players.

## Data Availability

All analysis codes are publicly available through our OpenNeuro dataset^31^. These include: EEG preprocessing scripts (\derivatives\preprocessed\preprocessing_code.m), SNR calculations (\derivatives\SNR\SNR_code.m), ERP analysis (\derivatives\ERP_analysis\ERP_code.m), time-frequency analysis (\derivatives\TF_analysis\TF_analysis_code.m), correlation analyses (\derivatives\correlation_analysis\correlation_analysis_code.m), and reliability assessments (\derivatives\reliability_analysis\reliability_analysis_code.m), ensuring full reproducibility of all reported results.

## References

[1] Pornpongtechavanich, P., Wuttidittachotti, P. & Daengsi, T. QoE modeling for audiovisual associated with MOBA game using subjective approach. *Multimed Tools Appl* **81**, 37763–37779, 10.1007/s11042-022-12807-1 (2022). 35493419 10.1007/s11042-022-12807-1 PMC9028896

[2] T’ng, S. T., Ho, K. H. & Pau, K. Need Frustration, Gaming Motives, and Internet Gaming Disorder in Mobile Multiplayer Online Battle Arena (MOBA) Games: Through the Lens of Self-Determination Theory. *Int J Ment Health Addict*, 1–21 (2022). 10.1007/s11469-022-00825-x 10.1007/s11469-022-00825-x PMC9037055 35497075

[3] Yang, H. B. & Zhang, X. Y. Revision and Evaluation of Diagnostic Efficiency for the Simplified Chinese Version of the Ten-Item Internet Gaming Disorder Test. *Studies of Psychology and Behavior* **21**, 658–666, 10.12139/j.1672-0628.2023.05.012 (2023).

[4] Gleich, T., Lorenz, R. C., Gallinat, J. & Kühn, S. Functional changes in the reward circuit in response to gaming-related cues after training with a commercial video game. *Neuroimage* **152**, 467–475, 10.1016/j.neuroimage.2017.03.032 (2017). 28323159 10.1016/j.neuroimage.2017.03.032

[5] Coronel-Oliveros, C. *et al*. Gaming expertise induces meso-scale brain plasticity and efficiency mechanisms as revealed by whole-brain modeling. *Neuroimage* **293**, 120633, 10.1016/j.neuroimage.2024.120633 (2024). 38704057 10.1016/j.neuroimage.2024.120633 PMC11875018

[6] Roy, S., Islam, M., Yusuf, M. S. U. & Jahan, N. EEG based stress analysis using rhythm specific spectral feature for video game play. *Comput Biol Med* **148**, 105849, 10.1016/j.compbiomed.2022.105849 (2022). 35870317 10.1016/j.compbiomed.2022.105849

[7] Mariman, J. J., Bruna-Melo, T., Gutierrez-Rodriguez, R., Maldonado, P. E. & Burgos, P. I. Event-related (de)synchronization and potential in whole vs. part sensorimotor learning. *Front Syst Neurosci* **17**, 1045940, 10.3389/fnsys.2023.1045940 (2023). 37025165 10.3389/fnsys.2023.1045940 PMC10070693

[8] Bailey, K., West, R. & Anderson, C. A. A negative association between video game experience and proactive cognitive control. *Psychophysiology* **47**, 34–42, 10.1111/j.1469-8986.2009.00925.x (2010). 19818048 10.1111/j.1469-8986.2009.00925.x

[9] Foerster, F. R., Chidharom, M. & Giersch, A. Enhanced temporal resolution of vision in action video game players. *Neuroimage* **269**, 119906, 10.1016/j.neuroimage.2023.119906 (2023). 36739103 10.1016/j.neuroimage.2023.119906

[10] Foerster, F. R., Chidharom, M., Bonnefond, A. & Giersch, A. Neurocognitive analyses reveal that video game players exhibit enhanced implicit temporal processing. *Commun Biol* **5**, 1082, 10.1038/s42003-022-04033-0 (2022). 36221032 10.1038/s42003-022-04033-0 PMC9553938

[11] Cui, R. *et al*. Co-activation patterns during viewing of different video game genres. *Brain Res Bull* **213**, 110974, 10.1016/j.brainresbull.2024.110974 (2024). 38710311 10.1016/j.brainresbull.2024.110974

[12] Palaus, M., Marron, E. M., Viejo-Sobera, R. & Redolar-Ripoll, D. Neural Basis of Video Gaming: A Systematic Review. *Front Hum Neurosci* **11**, 248, 10.3389/fnhum.2017.00248 (2017). 28588464 10.3389/fnhum.2017.00248 PMC5438999

[13] Ko, C. H. *et al*. Heterogeneity of gaming disorder: A clinically-based typology for developing personalized interventions. *J Behav Addict*10.1556/2006.2023.00059 (2023). 37934288 10.1556/2006.2023.00059 PMC10786230

[14] Na, E. *et al*. The influence of game genre on Internet gaming disorder. *J Behav Addict* **6**, 1–8, 10.1556/2006.6.2017.033 (2017). 28658960 10.1556/2006.6.2017.033 PMC5520129

[15] Balhara, Y. P. S., Singh, S. & Gupta, P. K. What Constitutes ‘Gaming’ in the Gaming Disorder?: Observations and Recommendations. *Indian J Psychol Med* **45**, 297–303, 10.1177/02537176221150601 (2023). 37152383 10.1177/02537176221150601 PMC10159567

[16] Long, K., Zhang, X., Wang, N. & Lei, H. Event-related prefrontal activations during online video game playing are modulated by game mechanics, physiological arousal and the amount of daily playing. *Behav Brain Res* **469**, 115038, 10.1016/j.bbr.2024.115038 (2024). 38705282 10.1016/j.bbr.2024.115038

[17] Klasen, M. *et al*. Selective reward responses to violent success events during video games. *Brain Struct Funct* **225**, 57–69, 10.1007/s00429-019-01986-7 (2020). 31754792 10.1007/s00429-019-01986-7

[18] Xi, W. & Hu, Y. Z. Internet Gaming Disorder in Adolescents:Review and Prospect. *Chinese Journal of Applied Psychology* **28**, 3–19, 10.3969/j.issn.1006-6020.2022.01.001 (2022).

[19] Wang, Z. L. *et al*. Gender-related differences in involvement of addiction brain networks in internet gaming disorder: Relationships with craving and emotional regulation. *Prog Neuropsychopharmacol Biol Psychiatry* **118**, 110574, 10.1016/j.pnpbp.2022.110574 (2022). 35569619 10.1016/j.pnpbp.2022.110574

[20] Pallavicini, F., Pepe, A. & Mantovani, F. The Effects of Playing Video Games on Stress, Anxiety, Depression, Loneliness, and Gaming Disorder During the Early Stages of the COVID-19 Pandemic: PRISMA Systematic Review. *Cyberpsychol Behav Soc Netw* **25**, 334–354, 10.1089/cyber.2021.0252 (2022). 35639118 10.1089/cyber.2021.0252

[21] Nuyens, F. *et al*. Impulsivity in Multiplayer Online Battle Arena Gamers: Preliminary Results on Experimental and Self-Report Measures. *J Behav Addict* **5**, 351–356, 10.1556/2006.5.2016.028 (2016). 27156376 10.1556/2006.5.2016.028 PMC5387787

[22] Sheehan, D. V. *et al*. The Mini-International Neuropsychiatric Interview (M.I.N.I.): the development and validation of a structured diagnostic psychiatric interview for DSM-IV and ICD-10. *J Clin Psychiatry* **59** Suppl 20, 22–33;quiz 34–57 (1998). 9881538

[23] Pontes, H. M., Király, O., Demetrovics, Z. & Griffiths, M. D. The conceptualisation and measurement of DSM-5 Internet Gaming Disorder: the development of the IGD-20 Test. *PLoS One* **9**, e110137, 10.1371/journal.pone.0110137 (2014). 25313515 10.1371/journal.pone.0110137 PMC4196957

[24] Qin, L. X., Liu, Q. S. & Luo, T. Reliability and Validity of 20-item Internet Gaming Disorder Test for Chinese College Students. *Chinese Journal of Clinical Psychology* **28**, 33–36, 10.16128/j.cnki.1005-3611.2020.01.008 (2020).

[25] Patton, J. H., Stanford, M. S. & Barratt, E. S. Factor structure of the Barratt impulsiveness scale. *J Clin Psychol* **51**, 768–774 (1995). 10.1002/1097-4679(199511)51:6<768::aid-jclp2270510607>3.0.co;2-1. 8778124 10.1002/1097-4679(199511)51:6<768::aid-jclp2270510607>3.0.co;2-1

[26] Li, X. Y. *et al*. Reliability and validity of an adapted Chinese version of Barratt Impulsiveness Scale. *Chinese Mental Health Journal* **25**, 610–615, 10.3969/j.issn.1000-6729.2011.08.013 (2011).

[27] Wang, L., Liu, H. C., Du, W. & Li, Z. Q. Test of Difficulties in Emotion Regulation Scale in Chinese People. *China Journal of Health Psychology* **15**, 336–340, 10.3969/j.issn.1005-1252.2007.04.019 (2007).

[28] Gratz, K. L. & Roemer, L. Multidimensional assessment of emotion regulation and dysregulation: Development, factor structure, and initial validation of the difficulties in emotion regulation scale. *Journal of psychopathology and behavioral assessment* **26**, 41–54 (2004).

[29] Gorgolewski, K. J. *et al*. The brain imaging data structure, a format for organizing and describing outputs of neuroimaging experiments. *Sci Data* **3**, 160044, 10.1038/sdata.2016.44 (2016). 27326542 10.1038/sdata.2016.44 PMC4978148

[30] Pernet, C. R. *et al*. EEG-BIDS, an extension to the brain imaging data structure for electroencephalography. *Sci Data* **6**, 103, 10.1038/s41597-019-0104-8 (2019). 31239435 10.1038/s41597-019-0104-8 PMC6592877

[31] Li, H. Z. *et al*. Research data supporting ‘EEG recording during playing MOBA game’. *OpenNeuro*10.18112/openneuro.ds005520.v1.0.1 (2024).

[32] Delorme, A. & Makeig, S. EEGLAB: an open source toolbox for analysis of single-trial EEG dynamics including independent component analysis. *J Neurosci Methods* **134**, 9–21, 10.1016/j.jneumeth.2003.10.009 (2004). 15102499 10.1016/j.jneumeth.2003.10.009

[33] Dong, L. *et al*. MATLAB Toolboxes for Reference Electrode Standardization Technique (REST) of Scalp EEG. *Front Neurosci* **11**, 601, 10.3389/fnins.2017.00601 (2017). 29163006 10.3389/fnins.2017.00601 PMC5670162

[34] Pion-Tonachini, L., Kreutz-Delgado, K. & Makeig, S. ICLabel: An automated electroencephalographic independent component classifier, dataset, and website. *Neuroimage* **198**, 181–197, 10.1016/j.neuroimage.2019.05.026 (2019). 31103785 10.1016/j.neuroimage.2019.05.026 PMC6592775

